# Water source of artificial plants in the northeastern margin of Tengger Desert based on hydrogen and oxygen stable isotopes

**DOI:** 10.3389/fpls.2025.1523085

**Published:** 2025-05-05

**Authors:** Zhenyu Zhao, Guodong Tang, Jinrong Li, Meng Wu, Lei Zhang

**Affiliations:** ^1^ Yinshanbeilu Grassland Eco-hydrology National Observation and Research Station, China Institute of Water Resources and Hydropower Research, Beijing, China; ^2^ Institute of Water Resources for Pastoral Area, Ministry of Water Resources, Hohhot, China; ^3^ Wuhai City Water Bureau, Inner Mongolia Autonomous Region, Wuhai Soil and Water Conservation Workstation, Wuhai, Inner Mongolia, China

**Keywords:** Tengger Desert, aerial seeding, dominant shrubs, hydrogen and oxygen isotopes, water source

## Abstract

Understanding water sources and utilization strategies is essential for the water use patterns of vegetation restoration species and achieving sustainable vegetation restoration. The water use strategies of *Corethrodendron scoparium* and *Calligonum mongolicum* were studied in the afforestation area on the northeastern edge of the Tengger Desert, to provide scientific guidance for regional vegetation restoration and stand structure adjustment. We utilize hydrogen and oxygen isotope techniques and the MixSIAR model to calculate the contribution rates of these two plant species to various potential water sources from June to October. By calculating the *PS* index, we determined the competitive relationship of *C. scoparium* and *C. mongolicum* towards different water sources. The results showed that the soil moisture content in the 0-80 cm soil layer changed significantly due to rainfall and evaporation, but stabilized with increasing depth. Shallow soil water shows enriched stable isotope composition, while the isotope of groundwater is relatively stable, and the isotope of precipitation is more enriched than that of groundwater. The main water source of *C. scoparium* and *C. mongolicum* was soil moisture. The utilization rate of 0-40 cm soil layer was 27% (*C.scoparium*) and 33% (*C.mongolicum*), and the utilization rate of 40-80 cm soil layer was 32% (*C.scoparium*) and 25% (*C.mongolicum*). The average proportional similarity index (*PS* index) between the two species was 95.67%, indicating a competitive relationship with water resources. When the surface layer (0-40) soil moisture is high (July, August), both species preferentially absorb water from this layer, and the water competition is reduced. The average *PS* index is 89%. When the surface layer (0-40) was deficient in soil moisture (June, September and October), the water competition increased, and the *PS* index was 97.83%. This study emphasized the adaptation strategies of these shrubs to arid environments and found that it provided key insights for optimizing vegetation density and species composition in desert aerial seeding areas and ensuring sustainable ecological restoration in the study area.

## 1 Introduction

Water is the key factor limiting the growth of artificial vegetation in arid and semi-arid regions (Chen et al., 2014). The availability and utilization patterns of water by plants reflect the ecosystem’s response to environmental water conditions, with eco-hydrological processes playing a decisive role in the function and evolutionary trajectory of the soil-vegetation system (Beyer et al., 2018). Therefore, studying plant water use is crucial for understanding the survival adaptability of species in aerial-seeding afforestation areas and for selecting sand-stabilizing vegetation. Since aerial seeding alters the original water balance and the spatial distribution of soil moisture in sandy areas, determining the mechanisms of water use by plants in arid and semi-arid regions is essential for the construction of artificial vegetation in these areas (Wang et al., 2017).

Stable isotope techniques are an important method for accurately determining the sources and proportions of water used by plants (Ehleringer and Dawson, 1992; Renée Brooks et al., 2009; Guillaume et al., 2014; David et al., 2017). By comparing the hydrogen and oxygen stable isotope values in the xylem water of plant stems with those of different water sources, these techniques provide insights into the water-use relationships of plants. In studies of plant water utilization, plants of different life forms generally utilize water from different sources, which is related to their root system types. Typically, trees and deep-rooted shrubs can access deep soil water or groundwater (Wei et al., 2013), while shallow-rooted shrubs and perennial herbaceous plants rely on shallow or intermediate soil water (Liu et al., 2011). In areas with higher precipitation, plants tend to use shallow soil water (Chen et al., 2016); in drier climates, they are more inclined to use deeper soil water and groundwater (Zhao et al., 2008). De Wispelaere et al. (Lien et al., 2017) studied seasonal variations in the water sources of plants in arid and semi-arid regions, finding that plants mainly use deep soil water and groundwater during the dry season, while relying on rainfall during the wet season. Dai et al (Dai et al., 2015). analyzed the water use patterns of *Haloxylon ammodendron* and *Haloxylon persicum* naturally distributed on dune tops and interdune lowlands in the Gurbantunggut Desert, revealing that these plants primarily utilize deep soil water and groundwater. In summary, the current research on plant water sources mainly focuses on hilly woodlands, forests, deserts, and riparian forests. However, there are few reports on the differences in water sources of artificial plants in arid and semi-arid areas, especially in the aerial seeding afforestation area on the northeastern edge of the Tengger Desert.

The Tengger Desert is located in the arid and semi-arid area of the Inner Mongolia Autonomous Region in northern China. In the 1980 s, China began large-scale aerial seeding afforestation in the Tengger Desert. After more than 30 years of development, the vegetation coverage of the Tengger Desert has been effectively improved. It has greatly promoted the ecological restoration of the Tengger Desert and realized the transformation from the past ‘ sand into human retreat ‘ to the present ‘ human into sand retreat ‘. In 1992, the vegetation coverage of the aerial seeding afforestation area in the northeastern margin of the Tengger Desert increased from less than 5% to more than 20% on average after more than 30 years of restoration, mainly due to the formation of plant communities dominated by *C.scoparium* and *C.mongolicum* in the aerial seeding area. The regional landform has developed from the original mobile dunes to fixed or semi-fixed dunes. However, with the large-scale sand-fixing afforestation, the contradiction between supply and demand of soil moisture is increasing, and vegetation in some high-density areas is even degraded. In addition, with the warming of the climate, the seasonal drought in the Tengger Desert occurs frequently, and the drought and water shortage seriously threaten the ecological restoration and sustainable vegetation construction in the desert area. However, water is the main limiting factor for vegetation restoration in the northeastern margin of the Tengger Desert. Some sand control plants with unreasonable planting density increase water consumption. What is the change of soil moisture in the plant community formed on this basis? After aerial seeding afforestation, what are the water sources used by the main artificial plants in the sandy area during the growing season, and are there any differences in their water sources? What kind of water use relationship does the plant have and what kind of water use strategy is reflected? Is there a competitive relationship between the mixed mode of *C.scoparium* and *C.mongolicum* in the utilization of soil moisture in this area? These issues remain to be studied.

Therefore, in this study, from June to October 2023, in the 1992 aerial seeding area in the northeastern margin of the Tengger Desert in Alxa Left Banner, Alxa League, the meteorological elements such as precipitation and temperature were monitored by small weather stations, and the hydrogen and oxygen stable isotope composition of atmospheric precipitation, plant xylem water, groundwater and soil water were measured. The MixSIAR model was used to study the water source characteristics and water use relationship of *C.scoparium* and *C.mongolicum* in different months, so as to clarify the water use strategy of *C.scoparium* and *C.mongolicum*, analyze the competitive relationship between *C.scoparium* and *C.mongolicum* on soil water use, and master the water use strategy of aerial seeding plants in detail. It provides reference for vegetation construction and regional desertification control in Tengger Desert.

## 2 Materials and methods

### 2.1 Overview of the study area

**Figure 1:**
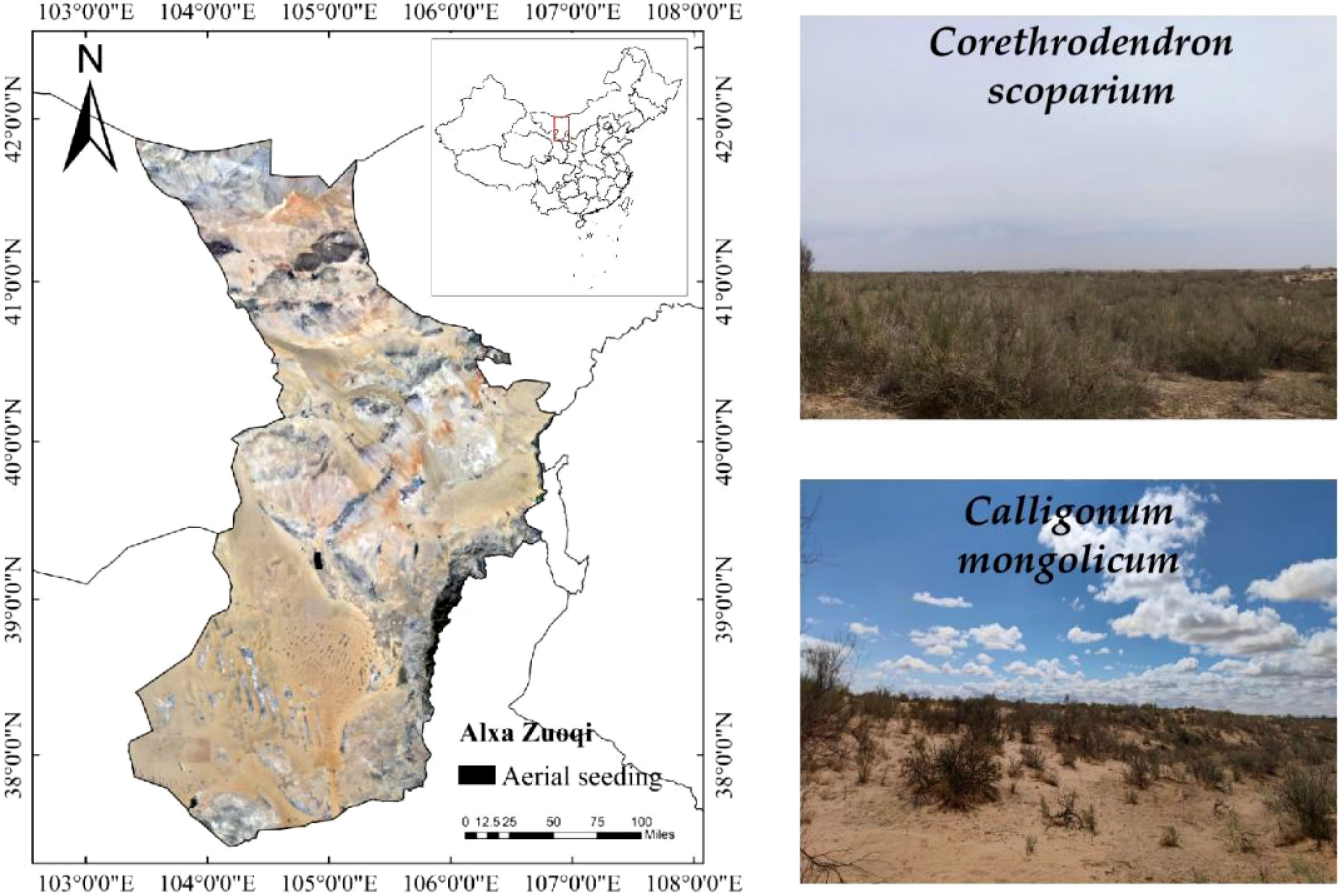
Geographic location of the study area.

The study area is located in the 1992 aerial-seeding afforestation zone on the northeastern edge of the Tengger Desert, Alxa Left Banner, Alxa League, as shown in **Figure 1**. The climate in the region is characterized by a mid-temperate continental climate. Although the area receives little rainfall, most of it is concentrated in July and August, with an annual average precipitation of only 123.33 mm. The total rainfall in June-October 2023 is 36.8 mm as shown in **Table 1**. The annual average temperature is 7.8°C, with extreme temperatures reaching up to 39°C. The average annual evaporation is 2258.8 mm, with a total of 3181 hours of sunshine per year, an average wind speed of about 4 m/s, and approximately 45 days of strong winds annually. The region is primarily dominated by the aerial seeding shrubs *C.scoparium* and *C.mongolicum*, along with the native shrub *Artemisia ordosica*. Herbaceous plants in the area include *Allium mongolicum*, *Stipa capillata*, *Grubovia dasyphylla*, and *Agriophyllum squarrosum*. The soil in the study area exhibits zonal distribution, including types such as gray desert soil, gray-calcic soil, and brown-calcic soil.

**Table 1:** Rainfall characteristics during the study period.

Month	Precipitation (mm)	Average temperature (°C)	Average wind speed (m/s)
June	4.8	23.8	3.72
July	18.5	25.3	3.81
August	7.6	24.8	3.14
September	4	19	3.36
October	1.9	10.1	2.64

### 2.2 Research methods

#### 2.2.1 Plot setup and investigation

The study area is located in the northeast edge of Tengger Desert of Alxa Left Banner in 1992. The study area was initially unvegetated. After more than 30 years of aerial seeding afforestation vegetation restoration, a vegetation pattern dominated by mixed plant communities of *C.scoparium* and *C.mongolicum* has been formed. From June to October 2023, a mixed plot of *C.scoparium* and *C.mongolicum* was selected in the study area. Firstly, the current vegetation status in the study area was investigated. The basic situation of different plant community samples is shown in **Table 2**. The survey indicators include plant height, crown width, base diameter, shrub coverage, and herbaceous plants. Based on the vegetation survey data, the standard growth parameters of representative plants were determined. Using these parameters as the standard, three healthy and representative strains of *C.scoparium* and *C.mongolicum* were selected for experimental sampling.

**Table 2:** Basic information of plant community.

Shrub composition	Plant height/cm	Tree crown /cm	Basal diameter /cm	Shrub cover/%	Herbaceous plant composition
*C.scoparium*	164 ± 12	174 ± 10	1.6 ± 0.2	21 ± 5	*Grubovia dasyphylla*, Stipa capillata, Agriophyllum squarrosum *Allium mongolicum Regel*
*C.mongolicum*	79 ± 24	93 ± 7	1.2 ± 0.4	19 ± 2	

#### 2.2.2 Sample collection

1. Plant Sample Collection: The experiment was conducted in June 2023. In different plots within the study area, healthy and mature *C.scoparium* and *C.mongolicum* plants were selected. Non-green lignified twigs (diameter 0.1-0.3 cm, length 3-5 cm) were clipped, peeled, and quickly placed into screw-top glass vials, which were then sealed with sealing film. The vials were immediately stored in portable ice boxes and transported back to the laboratory for freezing (below -20°C) for hydrogen and oxygen isotope analysis.
2. Soil Sample Collection: Soil samples were collected at half the canopy radius from where the plant samples were taken, at depths of 0-20 cm, 20-40 cm, 40-60 cm, 60-80 cm, 80-100 cm, 100-120 cm, 120-140 cm, 140-160 cm, 160-180 cm, and 180-200 cm. Three replicates were taken for each layer, with sampling times matching those of the plant samples. Each soil layer sample was divided into two parts: one part was quickly placed in sample bottles, sealed with sealing film, and stored in portable insulated boxes for transport to the laboratory for hydrogen and oxygen isotope value analysis. The other part was placed in aluminum boxes and brought back to the laboratory for soil moisture content measurement using the drying method.
3. Groundwater Sample Collection: Groundwater samples were taken from the nearest well in the study area.
4. The precipitation samples were taken from the standard rainfall buckets placed in the study area. The precipitation samples were collected immediately after each precipitation. The samples were sealed in a 10 ml glass bottle and brought back to the laboratory for stable isotope determination. Due to the scarcity of rainfall in the study area, only rainfall from 4 effective rainfall events can be collected.

#### 2.2.3 Water extraction and isotope determination

The processing and measurements in this study were conducted in the laboratory of the Institute of Water Resources for Pastoral Areas, Ministry of Water Resources. Plant and soil samples were processed using a low-temperature vacuum distillation system to extract water. The extracted water was then sealed and refrigerated for later analysis. The δ^18^O values of groundwater, precipitation, and the extracted water from plant xylem and soil were measured using a liquid water isotope analyzer from LGR. The δ value is widely used worldwide as a standard method to measure the number of hydrogen and oxygen stable isotopes, that is, the deviation of the isotope ratio in the sample from its reference (Vienna Standard Mean Sea Water) by one thousandth (Fry, 2015). The formula is (1):


(1) $$\delta \text{X}\left(‰\right)=\frac{R\text{sample}-R\text{standard}}{R\text{standard}}\times 1000$$

In the formula, δX is δD or δ^18^O (‰), *R* is the isotope ratio, *R*sample is the ratio of heavy and light isotope abundance of elements in the sample (^18^O/^16^O, D/H); *R*standard is the ratio of stable isotope abundance of international common standard (^18^O/^16^O, D/H stable isotope using VSMOW).

#### 2.2.4 Calculation of soil water content and the proportion of potential water sources used by plants

The soil water content was determined by drying method. The soil samples brought back to the laboratory were dried in a 105°C blast oven for 12 h, and the dry weight was weighed. The soil water content was calculated by the formula (Cao et al., 2018).


(2) $$W=\frac{W_{1}-W_{2}}{W_{2}-W_{0}}\times 100\%$$

In the formula, *W* is the mass water content (%), *W*_0_ is the weight of aluminum box, *W*_1_ is the weight of aluminum box + wet soil, and *W*_2_ is the weight of aluminum box and dried soil.

The MixSIAR model is a Bayesian mixing model that runs in R and can be used to determine the proportion of different potential water sources utilized by plants. It has the capability to handle any number of isotope values simultaneously, analyze multiple potential sources at once, operate quickly, and account for differences between isotope values. The Bayesian isotope mixing model MixSIAR estimates the proportion of potential water sources utilized by plants. It incorporates numerous factors related to multiple sources and their uncertainties, which enhances the accuracy and rigor of the model (Stock and Semmens, 2017). During model execution, the isotope values of the woody tissues of *C.scoparium* and *C.mongolicum* were input as mixing data into the MixSIAR model. The average values and standard deviations of isotope values from different soil depths (0-20, 20-40, 40-60, 60-80, 80-100, 100-120, 120-140, 140-160, 160-180, 180-200 cm) and groundwater were input as source data into the MixSIAR model. Since no isotope fractionation occurs during plant water uptake (Renée Brooks et al., 2009), the average values and standard deviations of potential sources were set to 0 as difference data for the MixSIAR model. The Monte Carlo Markov Chain run length was set to “short,” and the error term choice was set to “residual,” after which the model was executed (Wang et al., 2017).

#### 2.2.5 Proprtional similarity index

The proprtional similarity index (*PS*) is an indicator of niche overlap between organisms. The larger the *PS* index, the more the niche overlap between the two species, and the greater the competition for the same water source; the smaller the *PS* index, indicating that the niche overlap between the two species is not obvious, and the competition for the same resource is smaller (Stefan et al., 2016). In this study, *PS* index was used to evaluate the water use of *C.scoparium* and *C.mongolicum*. The specific calculation formula is shown in Formula 3:


(3) $$\text{PS}=1-0.5\sum_{\text{i}=1}^{\text{n}}\left|{\text{p}}_{1\text{i}}-{\text{p}}_{2\text{i}}\right|$$

In the formula: *P*_1_ *_i_* and *P*_2_ *_i_* represent the utilization ratio of the two plants to the i water source, respectively.

#### 2.2.6 Data analysis

This study used stable oxygen isotopes for analysis. Data were analyzed using SPSS 19.0, with one-way ANOVA employed to compare the significance of differences in soil moisture content across different soil depths and the δ^18^O values of woody tissue water. The MixSIAR model was used to analyze the contribution ratios of potential water sources to the plants. Graphs were created using Origin 8.0 software.

## 3 Results and analysis

### 3.1 Characteristics of soil water content

NOTE: Each graph represents the following sample plots for: (a) *C.scoparium*; (b) *C.mongolicum*; Different lowercase letters indicated that there were significant differences between different soil layers in the same plot (P< 0.05).

**Figure 2:**
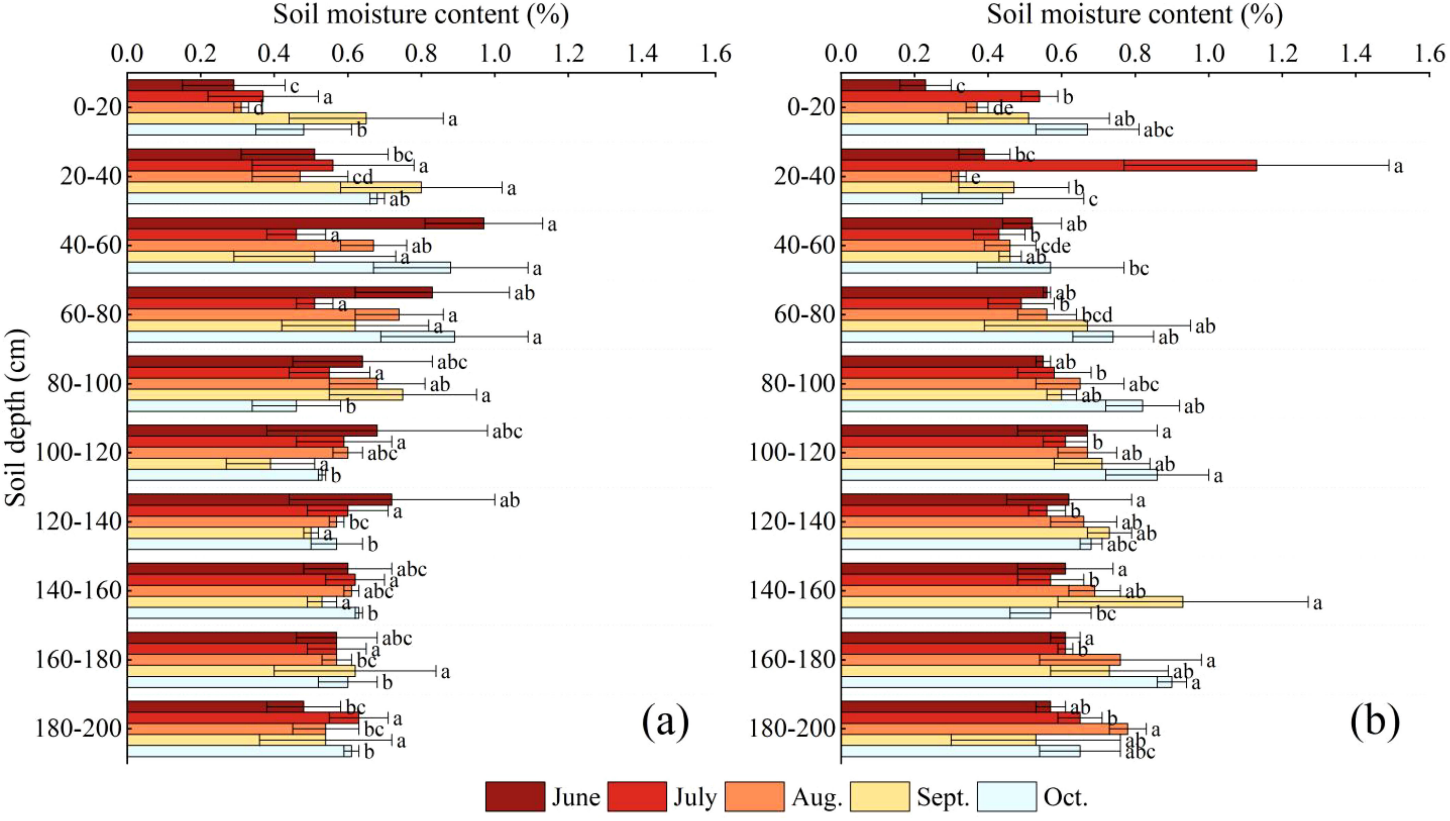
Variation characteristics of soil water content.

As shown in **Figure 2**, in general, the soil water content of *C.scoparium* community in the study area decreased first and then increased with the increase of month, and the soil water content of *C.mongolicum* community increased first, then decreased and then increased with the increase of month (Equation 1). In June, the change of soil water content was consistent with the change of soil depth. With the increase of soil depth, the water content increased first and then decreased. The soil water content of *C.scoparium* plants in 0-20 cm was relatively low, which was 0.29%, and the soil water content in 40-60 cm was higher, which was 0.97%. The soil water content of *C.mongolicum* plants in 0-20 cm was relatively low, which was 0.23%, and the soil water content in 100-120 cm was higher, which was 0.67%. In July, the soil water content of *C.scoparium* and *C.mongolicum* plants reached the maximum at 180-200cm and 20-40cm, respectively, 0.63% and 1.13%. In August, the soil water content of *C.scoparium* and *C.mongolicum* plants reached the maximum at 60-80cm and 180-200cm, respectively, 0.74% and 0.78%. In September, the soil water content of *C.scoparium* and *C.mongolicum* reached the maximum at 20-40cm and 140-160cm, respectively, 0.80% and 0.93%. In October, the soil water content of *C.scoparium* and *C.mongolicum* reached the maximum at 60-80cm and 160-180cm, respectively, 0.89% and 0.9%. The soil water content in different layers showed significant variations between the two species (*C.scoparium* and *C.mongolicum*) across different months (P< 0.05). For *C.scoparium*, the soil water content in the 0-20 cm layer was significantly lower than that in the 40-60 cm layer in June (P< 0.05). Similarly, for *C.mongolicum*, the soil water content in the 0-20 cm layer was significantly lower than that in the 100-120 cm layer in June (P< 0.05). In July, the soil water content in the 180-200 cm layer for *C.scoparium* and the 20-40 cm layer for *C.mongolicum* were significantly higher than other layers (P< 0.05). These results indicate that the two species exhibit distinct patterns of soil water utilization, with *C.scoparium* relying more on deeper soil layers during certain periods, while *C.mongolicum* shows a preference for shallower layers.

### 3.2 Hydrogen and oxygen stable isotope characteristics of different water sources

**Figure 3:**
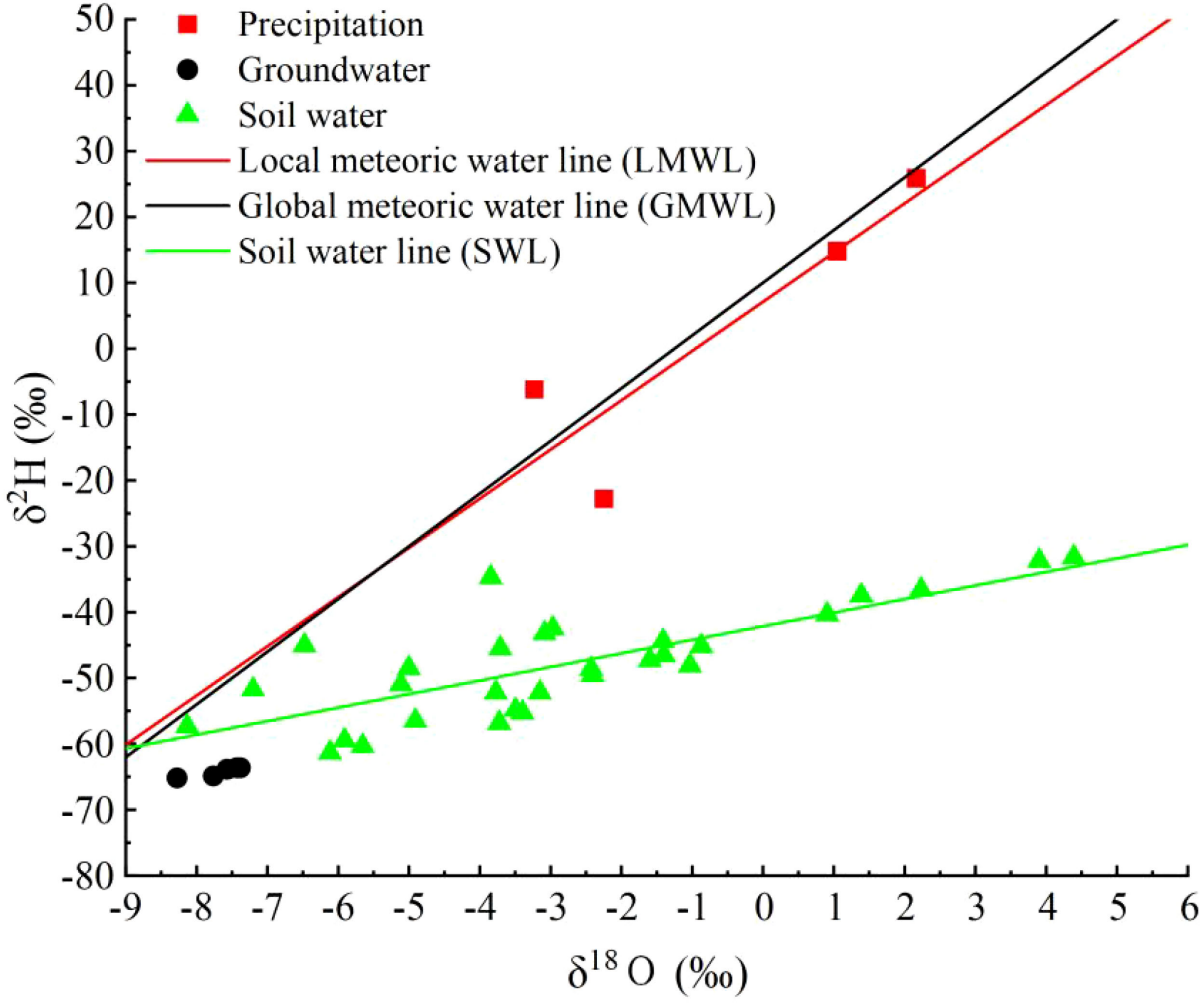
The distribution characteristics of atmospheric precipitation line, soil water line, precipitation and soil water δD and δ^18^O in the study area.

Based on the precipitation collected during the experimental period, the Local Meteoric Water Line (LMWL) for the study area was determined from the hydrogen and oxygen stable isotope values of the precipitation: δD = 7.47δ^18^O + 7.11 (*R*² = 0.79) (**Figure 3**). Its slope is lower than the Global Meteoric Water Line (GMWL): δD = 8δ^18^O + 10 (Craig, 1961), indicating that the precipitation in the study area experienced the effect of dry air during and produced secondary evaporation. The isotope variation range of the precipitation was: δ^18^O from -3.23‰ to 2.17‰ and δD from -22.82‰ to 25.88‰, with significant fluctuations. The isotope variation range of the groundwater was: δ^18^O from -8.28‰ to -7.38‰ and δD from -65.18‰ to -63.62‰, with smaller fluctuations. The isotope variation range of the soil water was: δ^18^O from -8.13‰ to 4.39‰ and δD from -61.36‰ to -31.68‰ (Equation 2). The Soil Water Line (SWL) equation was δD = 2.06δ^18^O - 42.13 (*R*² = 0.60), with both the slope and intercept lower than that of the LMWL, suggesting that the soil water was influenced by secondary evaporation.

The isotopic distribution characteristics of plant xylem water are shown in **Table 3**. For *C.scoparium*, the xylem water isotope range was: δ^18^O from 2.89‰ to 12.92‰ and δD from -45.11‰ to -32.21‰; for *C.mongolicum*, the xylem water isotope range was: δ^18^O from -0.09‰ to 8.62‰ and δD from -47.02‰ to -9.67‰. The monthly variations in xylem water isotopes for the two plants were as follows: for *C.scoparium*, the highest mean values of δD and δ^18^O were -32.21‰ and 12.92‰, respectively, both occurring in August, while the lowest values were -45.11‰ and 2.89‰, both in October; for *C.mongolicum*, the highest δD and δ^18^O values were -9.67‰ and 8.62‰, both in September, while the lowest were -47.02‰ and -0.09‰, also in September. For *C.scoparium*, the δD and δ^18^O values showed significant differences between June and August (P< 0.05), with the highest values observed in August. For *C.mongolicum*, the δD and δ^18^O values exhibited significant differences between June and September (P< 0.05), with the highest values observed in September. The study found that the δD and δ^18^O values of groundwater samples collected in different months were relatively consistent, indicating that the hydrogen and oxygen stable isotope composition of groundwater in the study area is relatively stable. However, a comparison of hydrogen and oxygen isotopes between groundwater and precipitation showed significant differences, with precipitation isotope values being more enriched than groundwater, indicating that precipitation in the study area almost does not contribute to groundwater recharge.

**Table 3:** Distribution characteristics of δD and δ^18^O in xylem water of plants.

Plant	Month	δD	δ^18^O
*C.scoparium*	June	-41.82 ± 3.35a	6.72 ± 2.51a
	July	-37.77 ± 2.5a	7.39 ± 4.04a
	August	-32.21 ± 16.58a	12.92 ± 9.48a
	September	-43.26 ± 4.32a	3.72 ± 1.73a
	October	-45.11 ± 10.5a	2.89 ± 3.9a
*C.mongolicum*	June	-47.02 ± 4.5b	-0.09 ± 1.66b
	July	-22.18 ± 4.62ab	6.18 ± 2.38ab
	August	-37.9 ± 3.1ab	4.87 ± 0.66ab
	September	-9.67 ± 15.16a	8.62 ± 1.88b
	October	-30.39 ± 28.42ab	4.21 ± 7.84ab

After the same column of data, different lowercase letters indicate significant differences between different months (P< 0.05).

### 3.3 Sources of soil moisture in different soil layers

**Figure 4:**
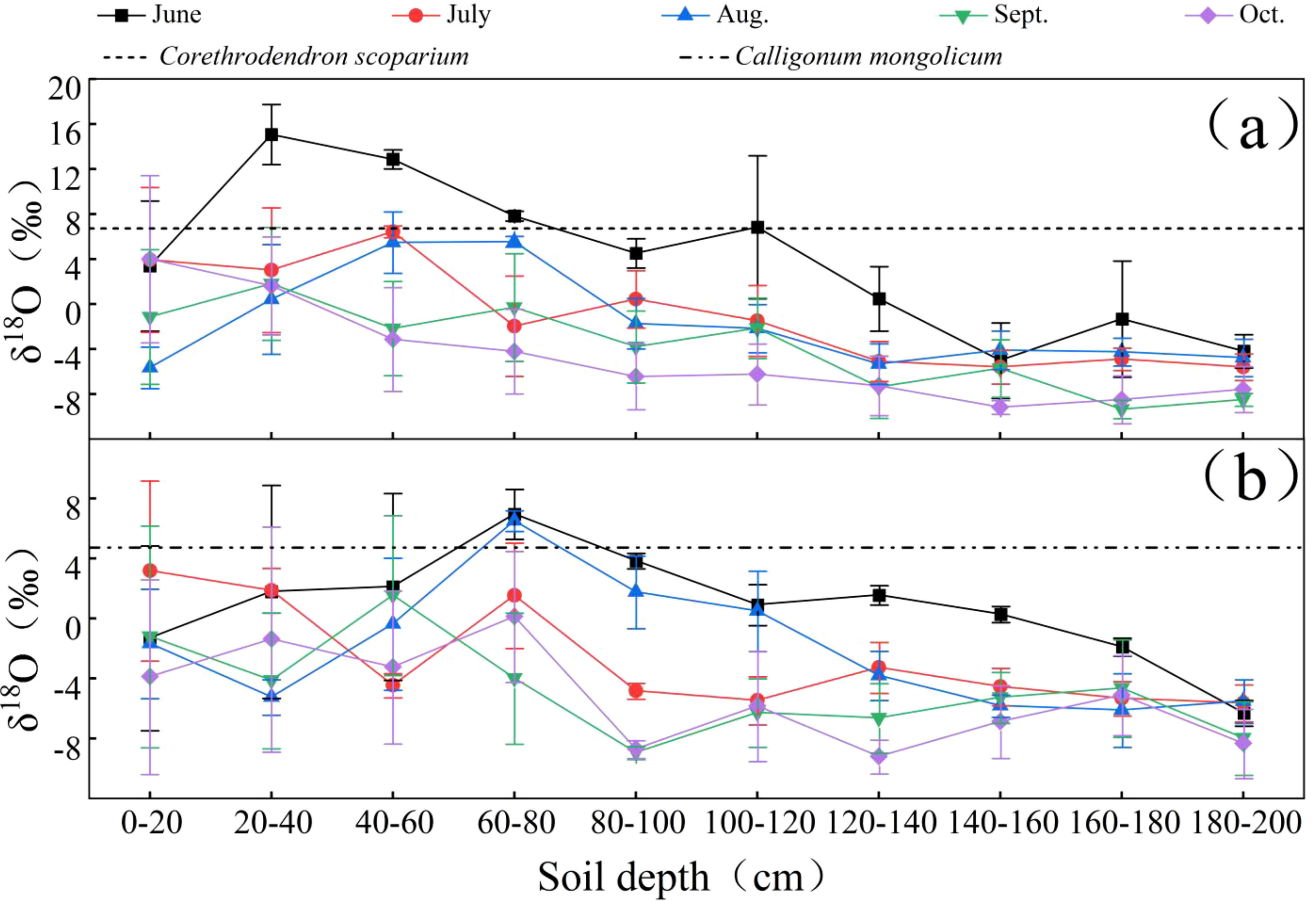
Comparison of δ^18^O values of soil water and plant xylem in different plots NOTE: Each graph represents the following sample plots for: **(a)** *C.scoparium*; **(b)** *C.mongolicum*.

As a stable isotope, δ ^18^O is very stable and is not prone to decay. In addition, δ ^18^O is able to record the movement process of water well, because the δ ^18^O value is used to study the soil water source. As shown in **Figure 4**, the δ^18^O values of soil water exhibit a decreasing trend with increasing soil depth. The potential water sources for the plants in the study area include shallow soil water (0-40 cm), upper soil water (40-80 cm), middle soil water (80-120 cm), lower soil water (120-160 cm), and deep soil water (160-200 cm). The soil layers that intersect with or have δ^18^O values closest to the δ^18^O values of the xylem water of *C.scoparium* and *C.mongolicum* represent the primary water uptake layers for these plants. Consequently, in June, *C.scoparium* primarily utilized shallow soil water, *C.mongolicum* primarily utilized upper and middle soil water. In July, *C.scoparium* primarily utilized upper soil water, *C.mongolicum* primarily utilized shallow soil water. In August, *C.scoparium* primarily utilized shallow and upper soil water, *C.mongolicum* primarily utilized upper and middle soil water. In September and October, *C.scoparium* primarily utilized shallow soil water, *C.mongolicum* primarily utilized shallow and upper soil water.

### 3.4 Analysis of water use sources of plants

**Figure 5:**
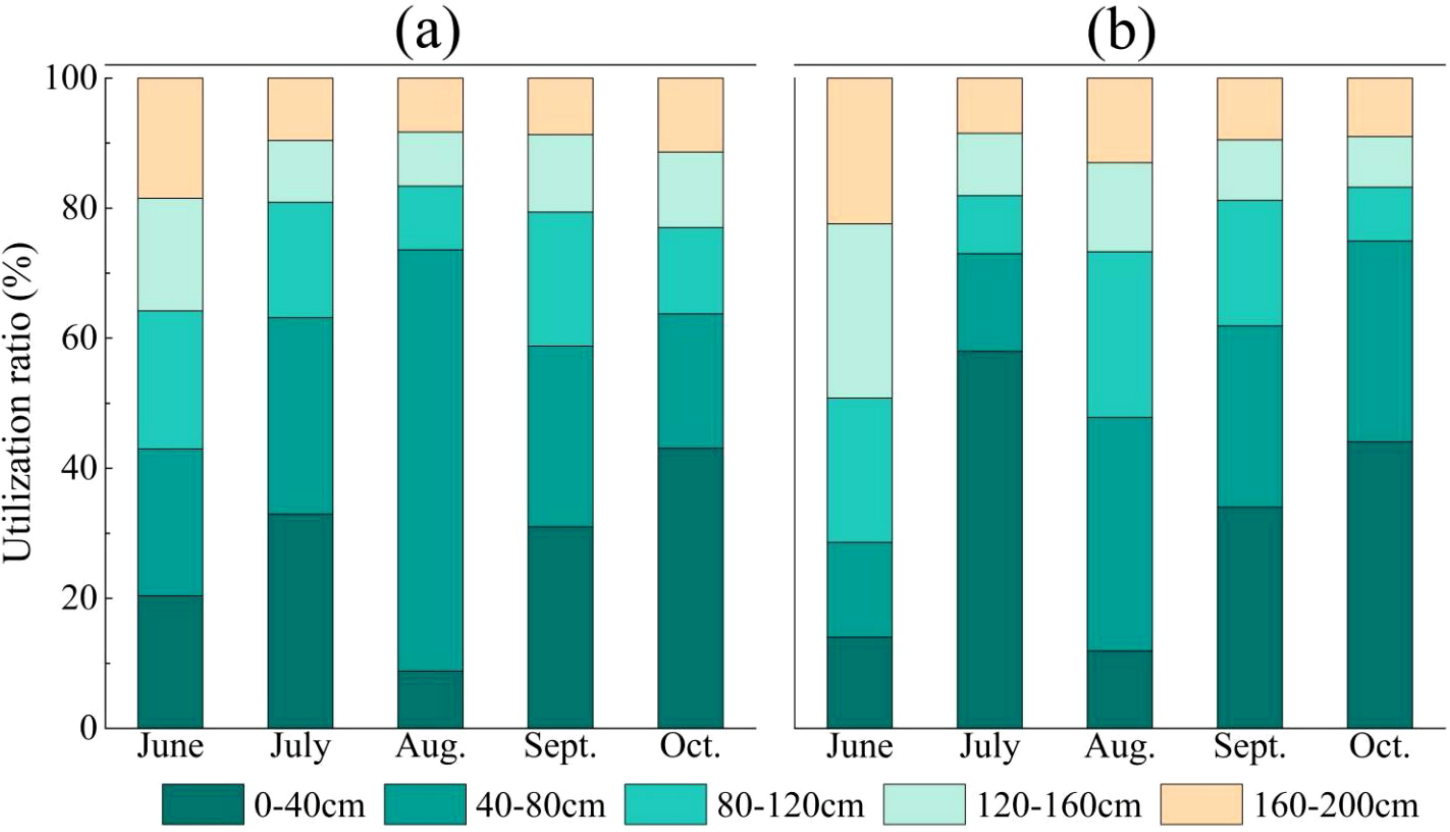
The utilization rate of potential water sources by two plants in different months NOTE: Each graph represents the following sample plots for: **(a)** *C.scoparium*; **(b)** *C.mongolicum*.

As shown in **Figure 5**, throughout the entire growing season, *C.scoparium* primarily utilized upper soil water, with a utilization rate of 33.2%. *C.mongolicum* mainly relied on shallow soil water, with utilization rates of 32.4%. The two species had the lowest utilization rates of deep soil water, at 11.3% and 12.5% respectively. However, the contribution rates of soil water from different layers to *C.scoparium* and *C.mongolicum* varied across different months. In June, which is outside the main growing season, 64.2% of the water absorbed by *C.scoparium* came from soil layers above 120 cm, mainly relying on shallow and upper soil water. *C.mongolicum* absorbed 71.4% of its water from soil layers below 80 cm, primarily depending on lower soil water. In the mid-growing season (July-August), *C.scoparium* absorbed 63.1%-73.5% of its water from soil layers above 80 cm, primarily relying on shallow soil water. *C.mongolicum* absorbed 47.8%-73% of its water from soil layers above 80 cm, mainly relying on shallow and upper soil water. In the late growing season (September-October), *C.scoparium* absorbed 58.8%-63.8% of its water from soil layers above 80 cm, mainly relying on shallow soil water. *C.mongolicum* absorbed 61.9%-75% of its water from soil layers above 80 cm, mainly relying on shallow soil water.

### 3.5 Competition relationship of plant water source

**Figure 6:**
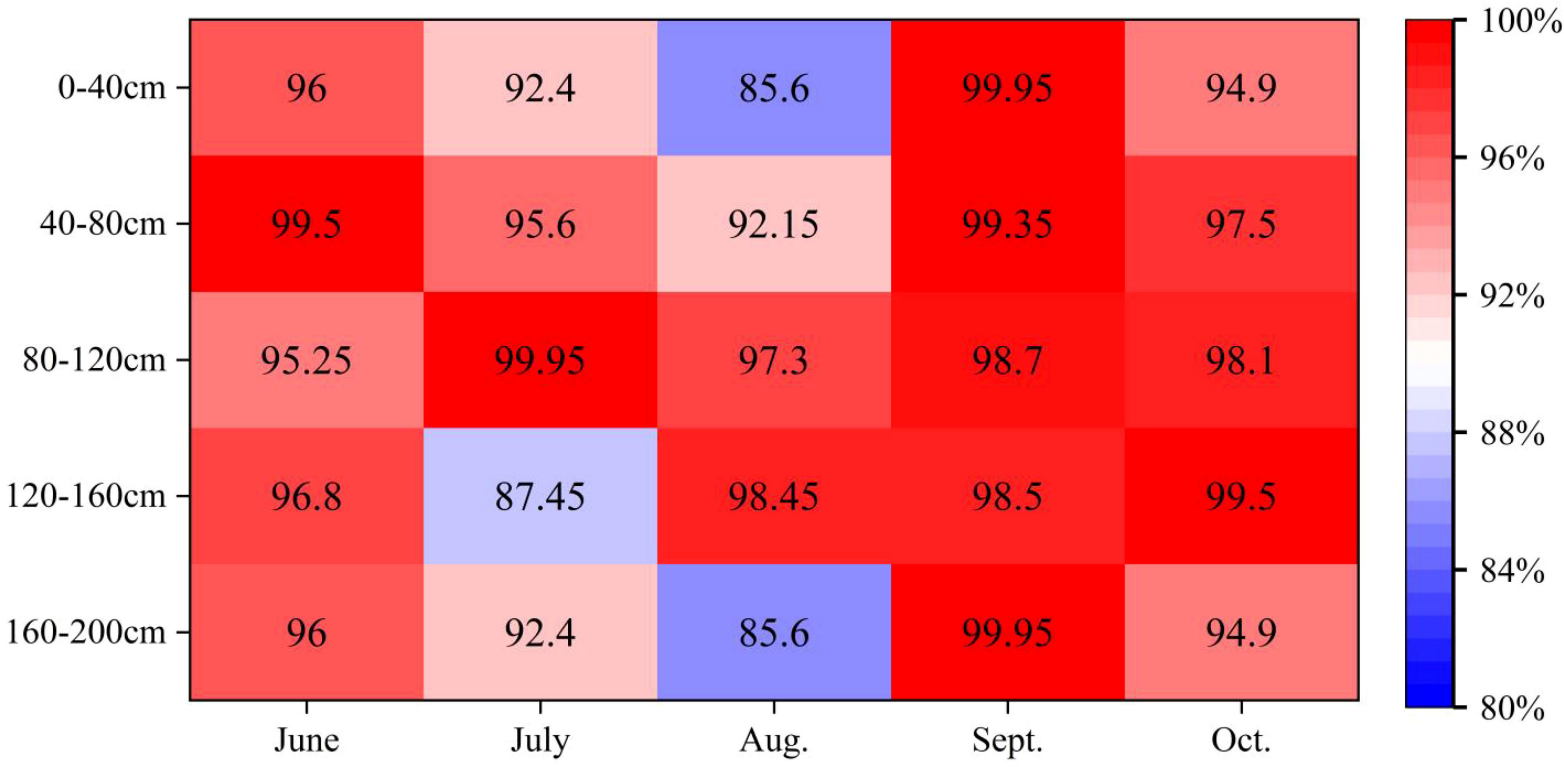
Comparison of *PS* index of two aerial seeding plants NOTE: *PS*: Proportional similarity index; *PS* = 0 indicates that there is no competition between the two plants. The *PS* index was between 0-100%, indicating that there was a competitive relationship between the two plants. And the larger the *PS* index, the stronger the competitive relationship; *PS* = 100% indicated that the competition between the two species reached the maximum.

In arid and semi-arid regions, plant competition for water resources is a common phenomenon. As shown in **Figure 6**, the *PS* index between the two plants is relatively high, which shows that *C.scoparium* and *C.mongolicum* have more use of the same water source (Equation 3). During the whole study period, the average *PS* index of *C.scoparium* and *C.mongolicum* was 95.67%, indicating that there was a competitive relationship between the water use of *C.scoparium* and *C.mongolicum*, but there were differences in the water use relationship between *C.scoparium* and *C.mongolicum* in different months. In September, the *PS* index of soil moisture in 0-200cm of *C.scoparium* and *C.mongolicum* was the highest, which was 99.29%, indicating that the water sources of *C.scoparium* and *C.mongolicum* were the same in September, the water use patterns were basically the same, and the water competition relationship was the strongest. The soil moisture *PS* index of 0-200 cm in August was the lowest, which was 91.82%, indicating that the main water sources of *C.scoparium* and *C.mongolicum* were different, the water use patterns were different, and the water competition relationship was relatively weak. In June, July and October, the *PS* index was smaller, indicating that the water use patterns of *C.scoparium* and *C.mongolicum* were different, and the water competition relationship was weak. At different depths, the *PS* indexes of 0-200 cm *C.scoparium* and *C.mongolicum* in June, September and October were all greater than 95%, indicating that the water competition relationship between *C.scoparium* and *C.mongolicum* was the strongest. In July 120-160 cm and August 0-40 cm, 160-200 cm *C.scoparium* and *C.mongolicum*, the *PS* indexes were 87.45%, 85.60% and 85.60%, respectively, which were lower than those in other soil layers, indicating that the water competition relationship between *C.scoparium* and *C.mongolicum* was the weakest.

## 4 Discussion

### 4.1 Soil moisture research

Soil moisture is primarily influenced by external factors such as evapotranspiration and atmospheric precipitation, with the impact of these external conditions gradually weakening as soil depth increases (Miriam et al., 2016; Shan et al., 2020). The soil water content in 0 ~ 60cm of *C.scoparium* and *C.mongolicum* plots was large and varied greatly. In the 100 ~ 200cm soil layer, the soil water content tends to be stable. This situation may be that the density of vegetation root system in deep soil is relatively small, and the root system absorbs less water from the soil. Because root absorption is one of the main ways of soil water consumption, the low root density leads to the relative reduction of deep soil water consumption, which helps to maintain the stability of water content. Compared with the surface soil, the deep soil is less affected by direct sunlight and air flow, so the evaporation is weaker. This further reduces the loss of deep soil moisture and helps to maintain a stable state of water content. Additionally, local climate variations, different vegetation types, and the combination of different soils directly affect the distribution of soil moisture. Differences in soil physical and chemical properties lead to heterogeneity in soil moisture (Miriam et al., 2016; Nicole et al., 2016).

### 4.2 Differences in plant water sources and water use relationship

Analysis of the relationship between atmospheric precipitation, plant water, soil water, and groundwater hydrogen and oxygen isotopes in the study area reveals that both the intercept and slope of the atmospheric precipitation equation are greater than those of the soil water equation. This indicates that precipitation primarily recharges soil water in the study area, while soil water is significantly affected by non-equilibrium evaporation. The minimal variation in groundwater hydrogen and oxygen isotopes suggests a stable isotopic composition, likely due to the deep burial of groundwater layers, which are less influenced by environmental factors compared to soil water. Consequently, the hydrogen and oxygen isotopes in the groundwater of the aerial seeding forestation area on the northeastern edge of the Tengger Desert are almost unaffected by evaporative fractionation. Due to their drought tolerance, resistance to sand burial, and rapid growth, *C.scoparium* and *C.mongolicum* have become important plants for sand control in the region in recent years. Moreover, plants are sensitive to changes in soil moisture and can adjust their water use strategies in time according to changes in soil moisture (Bush et al., 2010). The research findings indicate that during the growing season in the aerial seeding forestation area on the northeastern edge of the Tengger Desert, these plants primarily utilize soil water, with precipitation and groundwater contributing less than soil water. *C.scoparium* and *C.mongolicum* mainly rely on soil water in 0-40 cm and 40-80 cm soil layers, with a total contribution rate of more than 50%. The variation in soil water usage across different months is primarily influenced by rainfall; when rainfall is abundant, soil water is replenished, leading to changes in the plants’ soil water utilization. In August, when rainfall is higher, *C.scoparium* and *C.mongolicum* utilize soil water from the 40-80 cm layer, likely because rainfall recharges the soil water, allowing water to infiltrate and become available to plants at that depth. Wang et al (Wang et al., 2017, Wang et al., 2019). found that in *Vitex negundo*, water is mainly obtained from the surface soil layer (0-40 cm), and then shifts to the deep soil layer (120-300 cm) as the season progresses. Then the seasonal water use characteristics of *Spiraea pubescens* and *Hippophae rhamnoides* were investigated. The results showed that during the growing season, about 80% of the water source of *H.rhamnoides* and *S.pubescens* came from the 0-120 cm soil layer. Zhao et al (Zhao et al., 2021). studied the water use of *Salix psammophila* and *Caragana korshinskii* and found that the use of 0-30 cm soil water was shifted in July and August, with an average contribution rate of 61% and 51%, respectively. Ding et al (Ding et al., 2023). found that evergreen and deciduous tree species mainly use 0-50 cm soil water in summer. The above studies are similar to the results of this study. At the same time, we also found that plants tend to use it preferentially when the shallow soil moisture is sufficient (Hasselquist and Allen, 2009). It also shows that *C.scoparium* and *C.mongolicum* respond to different environments through spatial differences in water use.

### 4.3 Effects of plant roots on soil water use

Plant roots are one of the primary means by which plants absorb water, and they play a crucial role in determining the sources of water that plants utilize (Liu et al., 2011; Davi et al., 2012). We studied the root system. As shown in **Table 4**, the main roots of the two plants are distributed between 0-3m, while the lateral roots of the two plants are developed, mainly horizontally distributed between 0-1.5m, and the water absorption of the plants is mainly carried out by the lateral roots. Therefore, the utilization of water by plants is closely related to the distribution of roots. When the water conditions are different, the absorption of water by plant roots will also be different. In this study, *C.scoparium* mainly used 0-80 cm soil water from June to October, but the contribution rate of surface soil water was slightly higher. And *C.scoparium* mainly uses 0-40 cm soil water. The reason is that the lateral root system of *C.scoparium* is developed, and the lateral root grows horizontally (Ma et al., 2012). It will preferentially use 0-40 cm soil moisture, and the contribution rate to deep 120-200 cm soil moisture in June is higher than that in other months, indicating that in the period of drought or less rainfall, the developed main roots of *C.scoparium* will choose to use deeper soil moisture. The root system of *C.mongolicum* is developed, and the main root will grow to 2-3m, while the horizontal root system is mainly distributed in the surface and shallow layers. Therefore, *C.mongolicum* mainly uses 0-80cm soil water, and when the rainfall is scarce in June, it will also increase the use of soil water in other soil layers. This reflects the adaptive strategies of *C.scoparium* and *C.mongolicum* on water use. In plant-water systems in arid regions, plants have the ability to redistribute water absorbed by roots at different depths, so they can selectively absorb and utilize various types of water sources to meet their growth needs (Zeng and Ma, 2013).

**Table 4:** Root distribution characteristics of two plants.

Plant	Root depth (cm)	Root quantity	Base diameter (mm)	Root length density (m/m^3^)	Root biomass (g/m^3^)
*C.scoparium*	235 ± 88.46	44.33 ± 15.7	17 ± 4.58	3.32 ± 2.43	2736.21 ± 1505.54
*C.mongolicum*	186.67 ± 40.41	113 ± 100.46	14.33 ± 4.04	5.92 ± 7.47	3544.61 ± 2980.66

Nippert et al. (Jesse and Alan, 2007) found that when shallow soil water is insufficient to sustain normal plant growth, the root system of plants shifts from a shallow water uptake mode to a deeper water uptake mode. Consistent with our research findings, *C.mongolicum* utilizes soil water from the 120-160 cm and deep 160-200 cm layers during June when water availability is poor. However, in July and August, when rainfall is more abundant, the well-developed lateral roots of *C.mongolicum* prioritize the use of soil water from the 0-40 cm and 40-80 cm layers, reducing reliance on deeper water sources. This indicates that *C.mongolicum* is well-adapted to the soil moisture conditions in this region. The differing water uptake sources for the root systems of *C.scoparium* and *C.mongolicum* reflect their adaptation to the varying moisture conditions of the study area, showcasing the co-evolution of these two plants in this environment (Hao and Li, 2021). Therefore, the horizontal and vertical distribution of plant roots in the soil is one of the reasons for the differences in water uptake depth and sources among different plants.

### 4.4 Competition relationship of plant water source

The utilization of the same resources and the interspecific relationship between the two tree species under the same habitat conditions can be revealed by the niche overlap index. There are two possibilities for niche overlap between tree species: First, there is a competitive relationship between tree species sharing resources; second, there is a niche separation relationship between resource utilization among tree species, which includes temporal and spatial differences (Han et al., 2023; Li et al., 2024). In this study, *PS* index was selected as an index to characterize the niche overlap between plants. The study found that the *PS* index of the two plants was basically higher, indicating that there was a strong water competition between *C.scoparium* and *C.mongolicum* in most periods. This conclusion is similar to the results of (Tang et al., 2018). During the study period, the PS index of *C.scoparium* and *C.mongolicum* was higher. Combined with water use, both plants would use soil moisture in 0-80 cm soil layer. When the water competition between the two plants increased, *C.scoparium* would reduce the use of soil moisture in 0-80 cm soil layer, and *C.mongolicum* would increase the use of soil moisture in 0-80 cm soil layer. The variation characteristics of soil water uptake at different depths of the two species have similar responses to the competition of water sources between plants, which can be explained by niche separation. In September, the PS index of the two plants was higher than that in other months, indicating that the competition for soil moisture was the largest in September. The reason is that we speculate that under the condition of relatively high soil moisture content in September, due to the relative concentration of water resources and the strong demand for water by plants, coupled with niche overlap, root distribution conflict, soil texture and structural differences, the competition for water between plants will be fierce and complex. The degree of competition between the two plants for water sources is different in different periods, suggesting that this is an adaptation process of plants to changes in soil water conditions. At the same time, it shows that the two plants can achieve the purpose of coexistence through niche separation under continuous water resources competition.

## 5 Conclusions

The study investigated the water use strategies and competitive relationships of *C. scoparium* and *C. mongolicum* in the aerial afforestation area at the northeastern margin of the Tengger Desert.

1. Groundwater shows relatively stable hydrogen and oxygen stable isotopes, while precipitation isotopes are more enriched. Affected by precipitation and evaporation, stable isotopes of shallow soil moisture are enriched.
2. The main water source of *C. scoparium* and *C. mongolicum* was soil moisture, and the utilization rate was 45% and 41.1%, respectively. The water contribution rate of soil moisture in 0-80 cm soil layer to plant growth was the highest, reaching 60% and 57%, respectively. *C. scoparium* is more dependent on 40-80 cm soil moisture, and *C. mongolicum* is more dependent on 0-40 cm soil moisture.
3. The average proportional similarity index (*PS* index) between the two species was 95.67%, indicating a competitive relationship with water resources. When the surface layer (0-40) soil moisture is high (July, August), both species preferentially absorb water from this layer, and the water competition is reduced. The average *PS* index is 89%. When the surface layer (0-40) was deficient in soil moisture (June, September and October), the water competition increased, and the *PS* index was 97.83%.

## Data Availability

The original contributions presented in the study are included in the article/supplementary material. Further inquiries can be directed to the corresponding author.
